# Nutritional and Physical Rehabilitation in Post-Critical Coronavirus Disease 2019 (COVID-19) Ambulatory Patients: The NutriEcoMuscle Study

**DOI:** 10.3390/nu17101722

**Published:** 2025-05-20

**Authors:** Clara Joaquín, Irene Bretón, María Julia Ocón-Bretón, Alba Zabalegui, Diego Bellido, Pilar Matía Martín, Miguel Ángel Martínez-Olmos, Ana Zugasti, María Riestra, Francisco Botella, José Manuel García-Almeida

**Affiliations:** 1 Department of Endocrinology and Nutrition, Hospital Germans Trias i Pujol, Carretera Canyet, s/n, 08916 Badalona, Spain; 2 Department of Endocrinology and Nutrition, Hospital General Universitario Gregorio Marañón, C/Dr. Esquerdo, 46, 28007 Madrid, Spain; 3 Department of Endocrinology and Nutrition, Hospital Clínico Universitario Lozano Blesa, C/San Juan Bosco, 15, 50009 Zaragoza, Spain; mjocon@salud.aragon.es; 4 Nutritional Support Unit, Hospital Universitario Vall d’Hebron, Pg. de la Vall d’Hebron, 119 Horta-Guinardó, 08035 Barcelona, Spain; alba.zabalegui@vallhebron.cat; 5 Department of Endocrinology and Nutrition, Complejo Hospitalario Universitario de Ferrol (A Coruña), Av. da Residencia, s/n, 15405 Ferrol, Spain; diegobellido@gmail.com; 6 Endocrinology and Nutrition Department, Hospital Clínico San Carlos, Instituto de Investigación Sanitaria San Carlos (IdISSC), 28040 Madrid, Spain; pilar.matia@gmail.com; 7 Facultad de Medicina, Medicina II Department, Universidad Complutense de Madrid, 28040 Madrid, Spain; 8 Centro de Investigación Biomédica en Red de Diabetes y Enfermedades Metabólicas Asociadas (CIBERDEM), 28029 Madrid, Spain; 9 Department of Endocrinology and Nutrition, Complejo Hospitalario Universitario de Santiago de Compostela (CHUS), University of Santiago de Compostela (USC), 15706 Santiago de Compostela, Spain; miguel.angel.martinez.olmos@sergas.es; 10 Molecular Endocrinology Group, Instituto de Investigacion Sanitaria de Santiago de Compostela (IDIS), 15706 Santiago de Compostela, Spain; 11 CIBER Fisiopatologia de la Obesidad y Nutricion (CIBEROBN), 28029 Madrid, Spain; 12 Section of Clinical Nutrition and Dietetics, Complejo Hospitalario Universitario de Navarra, C/de Irunlarrea, 3, 31008 Pamplona, Spain; azugas@hotmail.com; 13 Department of Endocrinology and Nutrition, Hospital Universitario de Cabueñes, Los Prados, 395, 33394 Gijón, Spain; mriestra.fernandez@gmail.com; 14 Instituto de Investigación Sanitaria del Principado de Asturias (ISPA), 33011 Oviedo, Spain; 15 Nutrition Area coordinator, Spanish Society of Endocrinology and Nutrition, C/Villalar, 7, 28001 Madrid, Spain; botellaromero@gmail.com; 16 Department of Endocrinology and Nutrition, Hospital Virgen de la Victoria, H. Quironsalud. UMA, IBIMA Málaga, 29010 Málaga, Spain; jgarciaalmeida@gmail.com

**Keywords:** COVID-19, intensive care units, malnutrition, nutrition assessment, bioelectrical impedance analysis, nutritional ultrasonography

## Abstract

**Background**: The prevalence of malnutrition is high in post-intensive care unit (ICU) coronavirus disease 2019 (COVID-19) patients during hospitalization and after hospital discharge. This paper presents prospective results from the NutriEcoMuscle study, a multicenter observational study. The study aimed to evaluate changes in nutritional and functional status in post-ICU COVID-19 patients following nutritional and physical rehabilitation interventions. Secondary aims included assessing adherence to and tolerance of the oral nutritional supplement (ONS) used in the nutritional intervention. **Methods**: The study enrolled adults who had been admitted to the ICU due to severe COVID-19. At hospital discharge, the patients underwent a nutritional intervention based on oral nutritional supplements (ONSs) with 100% serum lactoprotein enriched with leucine and vitamin D and a physical rehabilitation program. They were followed up during three months. Performed assessments included Subjective Global Assessment (SGA), Global Leadership Initiative on Malnutrition (GLIM) criteria, Barthel index (BI), handgrip strength and Timed Up and Go test, bioelectrical impedance analysis (BIA), nutritional ultrasound (US), and tolerance and adherence to ONS. Sample size was calculated based on handgrip strength, and parametric and non-parametric tests were used to assess differences between the baseline and three-month outcomes. **Results**: The study included 96 patients (71.9% male, mean age 58.8 years, mean body mass index (BMI) of 28.8 kg/m^2^, 36.5% obese). A total of 85 patients (62 men and 23 women) completed the 90-day follow-up. The mean weight gain after the intervention was 6.8 (SD 5.2) kg (similar in men and women; *p* = 0.263). The proportion of patients with malnutrition according to the SGA or GLIM criteria decreased from 100% to 11.8% and 36.4%, respectively (*p* < 0.00001 in both cases). The proportion of patients with functional limitations by BI decreased from 66.7% to 27.0% (*p* < 0.0001). Handgrip strength increased more than 40% in both men and women (*p* < 0.00001). The time to perform the Timed Up and Go (TUG) test decreased more than 40% in both men and women (*p* < 0.00001). According to BIA, the mean fat mass did not increase significantly in either men or women. The mean fat-free mass index (FFMI) increased significantly in both men and women. There were also significant increases in body cell mass, skeletal muscle mass index, and appendicular skeletal muscle mass index. The phase angle (PhA) increased significantly in both men (26.5%) and women (17.4%). In a multivariate analysis, age and baseline PhA were related to the PhA increase (adjusted R^2^ = 0.5573). The US study showed a significant increase in the mean measurements of muscle area, muscle circumference, X-axis, and Y-axis in the rectus femoris. Regarding abdominal fat, there were no significant increases in total, superficial, or preperitoneal adipose tissue by US. Participants engaged in a median interquartile range (IQR) of 70 (0–120) min/week of strength exercise and 60 (0–120) min/week of moderate physical exercise. The supplement was well tolerated, and poor adherence (less than 50%) was low (4% of the participants). **Conclusions**: A three-month intervention, including ONS and physical rehabilitation, is associated with a significant improvement in nutritional and functional status. Patients gained weight primarily by increasing their muscle mass. There was no significant increase in fat mass, as measured by BIA or US. The intervention was well tolerated and had good adherence.

## 1. Introduction

The onset of the coronavirus disease 2019 (COVID-19) pandemic resulted in high rates of admissions to intensive care units (ICUs) (32%) and a high mortality rate of patients in ICUs (up to 39%) [1]. Severely ill patients often require prolonged stays in the ICU, which can last an average of 53 days, leading to nutritional and functional complications that warrant special attention [2,3]. Data from these patients may serve as a severe disease model and can be extrapolated to other pathologies that cause Acute Respiratory Distress Syndrome (ARDS) [4]. Early nutritional interventions in patients after severe respiratory COVID-19 can improve nutritional status, as measured by the Global Leadership Initiative on Malnutrition (GLIM) criteria [5,6], but the prevalence of undernutrition remains high, up to 66% three months after discharge [5]. Even one year after discharge, a significant proportion of post-ICU COVID-19 patients continue to experience low fat-free mass (FFM) and elevated fat mass despite overall improvements in weight and physical recovery [7]. In addition, sarcopenia is a major COVID-19 coexisting complication and is characterized by a decline in muscle strength and mass [8,9]. Sarcopenia and sarcopenic obesity are common in COVID-19 post-critical patients [10,11] and may still be present many months after hospital discharge despite standard nutritional support [12,13]. Moreover, the loss of muscle mass in post-critical COVID-19 ambulatory patients is difficult to assess due to technical limitations and the high prevalence of obesity [14,15].

The NutriEcoMuscle study was a multicenter study conducted in Spain that investigated the changes in functional status, nutrition, and body composition of patients treated for COVID-19 and discharged from the ICU [4]. Given the pandemic context and the urgent need to develop management strategies for post-ICU COVID-19 patients, this study was designed as an exploratory investigation. The study aimed to examine the impact of an intervention designed to aid muscle recovery and included taking an oral nutritional supplement (ONS) with 100% serum lactoprotein enriched with leucine and vitamin D, in combination with physical rehabilitation. The study used Bioelectrical Impedance Analysis (BIA) and nutritional ultrasound techniques to evaluate body composition. Nutritional ultrasound is a new non-invasive and portable technique for the evaluation of body composition, and provides detailed information about muscle mass and body fat [14,16,17]. Recently, the NutriEcoMuscle study published baseline results revealing that, upon hospital discharge, most COVID-19 patients who were critically ill experienced malnutrition and reduced muscle mass, leading to a loss of independence [4].

The current work describes the prospective phase of the NutriEcoMuscle study. The primary aim was to evaluate changes in nutritional and functional status, focusing on handgrip strength as a key indicator of muscle function recovery. Secondary aims included assessing adherence to and tolerance of the oral nutritional supplement (ONS) among post-ICU COVID-19 patients discharged from the hospital, as well as performing multivariable analyses to identify factors associated with changes in phase angle (PhA), a reliable marker of nutritional status. Hospital discharge serves as the baseline moment for the analysis. The patients underwent a 3-month recovery program, including an ONS designed to aid muscle recovery and physical rehabilitation.

## 2. Materials and Methods

The NutriEcoMuscle study was conducted in ten hospitals across Spain from March 2021 to January 2022. It included patients who were discharged from the hospital after being admitted to the ICU because of COVID-19. The patients underwent a nutritional and physical rehabilitation program and were followed up for three months.

This project was authorized by the Ethics Committee of the Hospital Universitari Germans Trias i Pujol (Badalona, Spain) under the code PI-20-321, approval date 13 November 2020. All procedures and materials used in this project followed the principles of the Declaration of Helsinki and the data protection and research regulations in Spain (Ley Orgánica 3/2018).

### 2.1. Participants

The study enrolled adults aged between 20 and 75 who were admitted to the ICU due to severe COVID-19 and had a hospital stay of more than 72 h. A COVID-19 diagnosis was confirmed using a positive SARS-CoV-2 real-time fluorescence polymerase chain reaction (RT-PCR) test. The management of ICU patients followed local clinical protocols. The exclusion criteria were as follows: pregnancy, patients with standing difficulties, patients with amputations, patients who had a Barthel index (BI) score of less than 60 (indicating severe dependency) before admission, patients with a previous body mass index (BMI) greater than 50 kg/m^2^, and patients who did not provide consent.

### 2.2. Intervention

At the baseline visit, all patients were provided with dietary recommendations and a specific ONS prescription for muscle recovery. The ONS was Fortimel^®^ Advanced (Nutricia, Danone, Madrid, Spain [18]). It is an oral supplement containing 100% whey protein (21 g), leucine (3 g), and vitamin D (10 µg), providing 302 kcal per serving (200 mL) [19]. The dosage of the product was two bottles per day. Table S1 (Supplementary Materials) provides detailed information about Fortimel^®^ Advanced [19]. The study funder (Nutricia) provided the products free of charge. Participants were also provided with specific exercise recommendations [20] and offered the possibility of carrying out a functional rehabilitation program. Nutritional and exercise assessments were conducted by professionals from each center’s nutrition and rehabilitation services, including dietitians and physical therapists. These professionals were responsible for evaluating both nutritional intake and exercise program adherence as part of the intervention described in the study. The exercise and muscle recovery program can be consulted at this link [20].

### 2.3. Variables

The previous article describes the collected variables, the techniques used, and the study’s baseline results [4]. In summary, the patients’ medical records provided demographic and clinical information, including age, Barthel index (BI) before hospitalization, length of hospital and ICU stay, co-morbidities, Sequential Organ Failure Assessment Score (SOFA), and whether or not they required orotracheal intubation. Informed consent was obtained at the time of hospital discharge, marking this prospective study’s baseline (time zero). At that moment, retrospective data from the hospital stay, including ICU admission, were collected. From this point onward, data were collected prospectively during the patient’s recovery period. All measurements followed a standardized protocol to ensure consistency and minimize variability.

Nutritional status was assessed using the Subjective Global Assessment (SGA) questionnaire [21], and malnutrition was diagnosed according to the GLIM criteria [22]. Nutritional intake was evaluated based on the proportion of their nutritional needs covered by their regular diet on days 0 and 90 (25%, 50%, 75%, or 100%). Functional status was evaluated using IB, handgrip strength, and the Timed Up and Go (TUG) test. Handgrip strength was measured on three separate occasions with the Jamar^®^ dynamometer (in the second handle position). The average of the three measurements was used for the analyses following the recommendations of the American Society of Hand Therapists [23]. The handgrip strength reference values used to diagnose dynapenia were the cutoffs recommended by the EWGSOP2 consensus (<16 kg for women and <27 kg for men) [24]. The TUG result was considered pathological if the patient needed more than 20 s to complete the test [25].

Different BIA devices were used depending on the hospital where the test was performed: BIA 101 BIVA (Akern, Pontassieve, Italy; www.akern.com, accessed on 10 April 2025); NUTRILAB (Akern, Pontassieve, Italy; www.akern.com, accessed on 10 April 2025); QUADSCAN 4000 (Bodystat, Douglas, Isle of Man, UK; www.bodystat.com, accessed on 10 April 2025); INBODY 770 (Inbody, Seoul, Republic of Korea, www.inbody.com, accessed on 10 April 2025); INBODY S10 (Inbody, Seoul, Republic of Korea, www.inbody.com, accessed on 10 April 2025); SECA 525 (Seca, Catalonia, Spain, www.seca.com/es, accessed on 10 April 2025); TANITA 780 (Tanita, Arlington Heights, IL, USA, www.tanita.com, accessed on 10 April 2025). The test was performed following the manufacturer’s instructions for each type of equipment, and all patients’ measurements were obtained using a 50 kHz phase-sensitive impedance analyzer. The assessed variables included the following: weight (kg), fat mass (kg), fat-free mass (FFM) (kg), FFM index (FMMI) (kg/m^2^), body cell mass (kg), skeletal muscle mass index (SMMI) (kg/m^2^), appendicular skeletal muscle mass (ASMM) index (kg/m^2^), PhA (degrees), standardized PhA (SPA), raw resistance (RZ) (ohmios), reactance (XC) (ohmios). The cutoff points for reduced muscle mass were established according to the guidelines for undernutrition (GLIM): FFMI < 17 for men and <15 kg/m^2^ for women; ASMM index < 7 kg/m^2^ for men and <5.7 kg/m^2^ for women [22]. Participants were classified as having a low PhA (<3.95°) based on a previous study involving COVID-19 patients [10]. This PhA cutoff point has been linked to higher mortality in individuals with COVID-19 [10]. The standardized PhA value was determined from the sex- and age-matched reference population value by subtracting the reference PhA value from the observed patient PhA, and then dividing the result by the respective age- and sex-reference standard deviation (SD); SPhA = [(measured PhA − mean population reference PhA)/SD of the reference population PhA] [10].

To perform the nutritional ultrasound assessments, all participating centers used a Mindray Z7 ultrasound machine manufactured by Shenzhen Mindray Bio-Medical Electronics Co., Ltd. in Shenzhen, China. The adipose tissue and musculoskeletal areas were evaluated with a 10–12 MHz soft tissue transducer and a multi-frequency linear array probe (probe width 40 mm). Rectus femoris muscle was evaluated with the patient in the supine position and with the transducer placed transversely at the lower 1/3 of the distance between the pelvis’s anterosuperior spine and the patella’s upper edge. Rectus femoris cross-sectional area (RFCSA), circumference, and longitudinal (*Y*-axis) and transverse (*X*-axis) distance measurements were obtained [16]. The *X*-axis represents the transverse (width) diameter of the rectus femoris muscle, measured from side to side. The *Y*-axis represents the anteroposterior (thickness) diameter, measured from front to back, both obtained in the axial (cross-sectional) plane with the probe placed perpendicular to the muscle. The measurement of adipose tissue thickness was established as the linear distance between the epidermis and the aponeurosis of the quadriceps rectus femoris [16]. For the evaluation of abdominal subcutaneous adipose tissue, the transducer was placed at the midpoint between the xiphoid process and the umbilicus, and the total, superficial, and preperitoneal adipose tissues were measured. The images were taken during unforced expiration, in a transverse axis, and with an alignment perpendicular to the skin. The visceral adipose tissue was determined by measuring the distance between the edge of the parietal peritoneum and the inner face at the junction of the two rectus abdominal muscles. To reduce interobserver variability, all investigators were trained to adhere to a specific nutritional ultrasound protocol before commencing the study [16].

The patients were assessed at the time of their hospital discharge (which served as the baseline) and after 90 days. Adherence and tolerability to the ONS were followed up by phone after 45 days. Table S2 (Supplementary Materials) provides detailed information about the workflow. Data on quality of life were collected as part of this study; however, the analysis and reporting of these results will be presented in a separate publication.

### 2.4. Sample Size

To calculate the sample size, handgrip strength was used as the primary endpoint, given its robustness as an indicator of muscle function recovery and its clinical relevance in post-ICU patients. Additionally, phase angle (PhA) and muscle ultrasound parameters were explored as complementary endpoints, as indicated in previous studies [26,27]. To consider a difference of at least 1 kg in the change in handgrip strength in patients before and after treatment as significant, assuming a standard deviation of 3.0, we estimated a required sample of 120 patients, with a 5% significance level and 90% power, accounting for a potential 20% loss to follow-up and/or dropout. Similarly, to consider a difference of 0.5 cm^2^ in the change in the rectus femoris muscle area as significant, assuming a standard deviation of 1.0, or a change of 0.2 cm in the X-axis with a standard deviation of 0.5 after three months of treatment, we required a sample of 83 patients, with a 5% significance level and 90% power, accounting for a potential 20% loss to follow-up and/or dropout. To consider a difference of 0.5 points in the change in PhA as significant, assuming a standard deviation of 1.5 after three months of treatment, we required a sample of 120 patients, with a 5% significance level and 90% power, accounting for a potential 20% loss to follow-up and/or dropout. Therefore, a total sample of 120 patients was needed to detect differences in muscle ultrasound, handgrip strength, and PhA before and after three months of treatment.

### 2.5. Statistical Analysis

The quantitative variables were expressed as mean and standard deviation or median and interquartile range. Frequencies and percentages were calculated for qualitative variables. Differences between qualitative variables were compared using the X2 or Fisher’s exact test, depending on the circumstance. Comparison of quantitative variables was performed by Student’s *t*-test (or Mann Whitney U-test, if conditions required). Correlations between percentage change in handgrip strength and TGU test results and percentage change in BIA and nutritional ultrasound measurements (and between these two techniques) were performed and analyzed by Spearman’s correlation coefficient.

A multivariate analysis was performed with a multiple linear regression model, with the percentage change in PhA as a dependent variable. The independent variables were those baseline clinical or epidemiological variables related to the percentage change in PhA with a statistical significance of *p* < 0.20 in the univariate study. Additionally, the model included the following baseline relevant clinical variables that the research team deemed necessary to include in the analysis despite their statistical significance: sex, vitamin D, C reactive protein (CRP), and history of diabetes mellitus. The assumptions of linear regression, including linearity, independence of errors, homoscedasticity, and absence of autocorrelation, were checked using the Durbin-Watson test. A *p*-value <  0.05 was considered statistically significant. All analyses were performed with Stata version 16.1 (StataCorp LLC, College Station, TX, USA).

## 3. Results

A total of 96 patients were included in the study. Table 1 displays the population’s demographic, clinical, and functional characteristics stratified by sex. Patients were predominantly males; the mean age (SD) was 58.8 (8.5) years. Thirty percent were over 65 years of age. The mean number of recorded co-morbidities was 1.1, and 50% of the population reported 0 to 2 pathologies. Obesity (41.7%) and high blood pressure (HBP) (35.4%) were the most prevalent co-morbidities. No significant differences were found between genders in demographic or clinical characteristics, except for obesity, where females had a significantly higher prevalence than males (*p* = 0.0386). At hospital discharge, most participants (66.7%) had some functional limitations after BI assessment. The results of handgrip strength indicated dynapenia in 62.5% of the cases (<16 kg for women and <27 kg for men). Regarding the TUG test, 20.3% of men and 44.4% of women had pathological results (>20 s). These differences were statistically significant (*p* = 0.0224).

A total of 85 patients (62 men and 23 women) completed the 90-day follow-up. The reasons for non-completion of the study were loss to follow-up (4 patients), investigator’s decision (2), patient’s decision (3), protocol violations (1), and death (1). The mean percentage of nutritional requirements that the patient met with the conventional diet was 75.5% (17.1%) at baseline and 94.1% (13.7%) at the end of the 90-day follow-up.

### 3.1. Nutritional Evaluation

Table 2 shows nutritional status based on weight, BMI, waist circumference, SGA, and GLIM criteria at baseline and after the intervention (stratification by sex can be found in Table S3, Supplementary Materials). The mean weight gain after the intervention was 6.8 (5.2) kg, with no significant differences between men and women (*p* = 0.263). There was a significant BMI increase in both men and women, which tended to be greater in men (9.8% vs. 1.8% increase; *p* = 0.061). Regarding waist circumference, there was a statistically significant increase of 5.5 (6) cm in men (*p* < 0.00001) and a non-significant increase of 0.18 (9.7) cm in women (*p* = 0.440).

**Figure 1:**
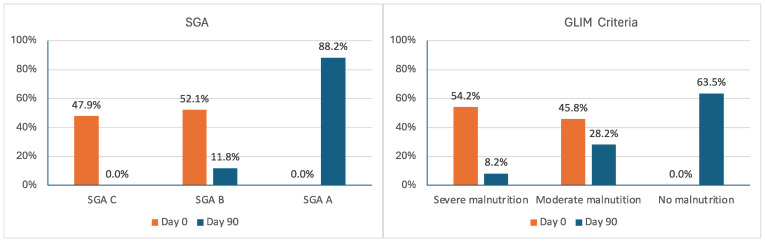
Changes in the proportion of malnourished participants according to the SGA and GLIM criteria.

The proportion of patients with malnutrition according to the SGA or GLIM criteria decreased from 100% to 11.8% and 36.4%, respectively (*p* < 0.00001 in both cases). The proportion of severe malnutrition decreased from 47.9% to 0% (*p* < 0.00001) by SGA (SGA C), and from 54.2% to 8.2% (*p* < 0.00001) by GLIM (Figure 1).

### 3.2. Functional Assessment

Table 3 shows the baseline functional status and changes after the intervention (stratification by sex can be found in Table S4, Supplementary Materials). At the end of the study, the proportion of patients with some functional limitation according to the BI decreased from 66.7% to 27.0% (*p* < 0.0001). This proportion was significantly lower in men compared to women (16.1% vs. 56.5%; *p* < 0.0001). The handgrip strength increased more than 40% in both men and women (*p* < 0.00001 in both cases). The proportion of patients with dynapenia was reduced by approximately 50% in both men and women. The time needed to perform the TUG test decreased by more than 40% in both men and women (*p* < 0.00001 in both cases). The proportion of patients with pathological TUG was reduced from 27.1% to 4.7% (*p* < 0.0001), with significant reductions in both men and women.

### 3.3. Body Composition Assessment

**Figure 2:**
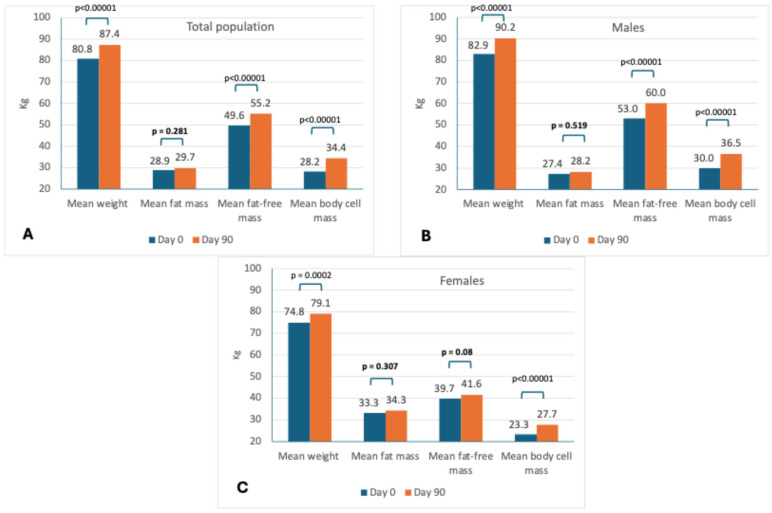
Main changes in body composition by BIA in the total population (**A**), males (**B**), and females (**C**).

Table 4 and Figure 2 provide information regarding BIA (stratification by sex can be found in Table S5, Supplementary Materials). Mean weight increased by 6.6 (4.8) kilograms. Mean fat mass did not increase significantly either in men or women. The mean FFMI increased significantly for both men and women. The increase was greater for men (23.9%) than women (7%). Low FFMI (<17 men and <15 kg/m^2^ women) was observed in 33.3% of the population at baseline, without differences between men and women. This proportion decreased significantly in men (35.8% to 14.2%; *p* = 0.0034) but not women (26.1% to 25%; *p* = 0.467).

There were significant increases in body cell mass (22.3% in men and 17.3% in women). There were also significant increases in SMMI in both men and women, but of greater magnitude in women (36.5%) than in men (12.3%). There was a similar magnitude of increase in the ASMM index in men (11.4%) and in women (10.0%), although with n = 9 at the end of the study, the change was not statistically significant in women. The mean PhA increased significantly in both men and women, and the proportion of patients with PhA < 3.95° decreased in both groups (*p* = 0.061 for women).

**Figure 3:**
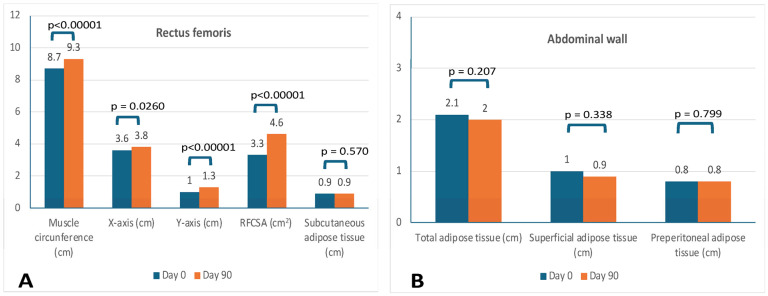
Changes in nutritional ultrasound assessments in rectus femoris (**A**) and abdominal wall (**B**). Abbreviations: RFCSA, rectus femoris cross-sectional area.

Table 5 and Figure 3 provide information regarding nutritional US. There was a significant increase in the mean measures of the RFCSA and *Y*-axis in rectus femoris assessment for both men and women. Men also experienced a significant increase in the *X*-axis and muscle circumference. There were no significant changes in rectus femoris adipose subcutaneous tissue. Regarding the assessment of abdominal fat, there were no significant increases in the mean measures of total adipose tissue, superficial adipose tissue, or preperitoneal adipose tissue. A statistically significant decrease of 9.3 (21.1)% in mean total adipose tissue was observed in women.

Table 6 presents the correlation between baseline variables and the percentage changes in PhA through univariate analysis. The percentage increase in PhA was lower in older patients, particularly in women, and in patients with diabetes, as well as in those with a higher mean handgrip strength or higher PhA at baseline. On the other hand, the increase was greater in those with a longer hospital stay, longer ICU stay, and worse results in the TUG test. In a multivariate analysis, age (beta: −1.2; 95% CI: −2.3 to −0.69; *p* = 0.039) and baseline PhA (beta: −15.9; 95% CI: −28.6 to −3.2; *p* = 0.017) were variables statistically and inversely related to the increase in PhA (adjusted R2 = 0.5573).

### 3.4. Correlations Between Changes in BIA and Nutritional Ultrasound

Percentage changes in muscle-related variables, such as circumference and *Y*-axis, as well as total and preperitoneal adipose tissue, significantly correlated with percentage weight and fat mass gain by BIA. Percentage changes in body cell mass correlated directly with percentage changes in RFCSA and *X*-axis and percentage changes in the ASMM index with percentage changes in RFCSA and *Y*-axis (Table S6; Supplementary Materials).

### 3.5. Correlations Between Changes in BIA and Nutritional Ultrasound, and Changes in Handgrip Strength and TUG

The percentage changes in handgrip strength correlated significantly with the percentage changes in PhA and three muscle parameters (RFCSA, *X*-axis, and *Y*-axis) but not with total, superficial, or preperitoneal abdominal fat. Percentage changes in TUG correlated inversely with SSMI and PhA by BIA and *Y*-axis on nutritional ultrasound (Table S7, Supplementary Materials).

### 3.6. Supplement Adherence and Tolerance

After 90 days of follow-up, 68 out of the 85 patients who completed the study (80%) maintained the prescribed initial dose of ONS. The median dose taken per day was 400 mL (IQR 200–400), which was the same for men and women. A total of 11 out of 85 patients (12.9%) temporarily discontinued the treatment during the study. In six cases, this was because of adverse events (AEs). Mean adherence to treatment was 82.6 (31)% with regards to the first dose and 77.0 (35.6)% regarding the second dose. Median adherence was 100% (IQR 75–100) with regards to the first dose and 100% (IQR 50–100) regarding the second dose, and it was similar in men and women. In total, 70.85% and 63.5% of the 85 patients had 100% adherence to the first and second prescribed doses, respectively, and 4% had poor adherence (<50%). No significant differences were observed in adherence rates (> or <70%) based on sex, age (>65), or diabetes status.

Table S8 (Supplementary Materials) shows the adverse events experienced during treatment. At days 45 and 90 of follow-up, 28.8% and 21.1% of patients reported experiencing at least one AE. Mild bloating and flatulence were the most frequently mentioned AEs. All gastrointestinal symptoms had a median score of 0, with the exception of feeling full after taking the ONS, with a median score of 5 (IQR 2–8).

On a scale of 0 to 10 (0: very bad–10: very good), 78.8% at day 45 and 82.3% at day 90 of follow-up reported an overall situation score of seven or higher.

### 3.7. Exercise

The median IQR estimated exercise time of each patient who completed the study was as follows: strength exercise: 70 (0–120) min/week; moderate physical exercise: 60 (0–120) min/week; walking: 210 (120–420) min/week; steps per day: 5300 (3500–8800). In total, 59 out of 85 (69.4%) patients followed a functional rehabilitation program. There were no differences in the performed exercises between men and women except for strength exercises, with a median of 95 (30–150) min/week in men and 30 (0–100) min/week in women (*p* = 0.0281).

## 4. Discussion

This multicenter, observational study conducted across ten hospitals in Spain aimed to explore the potential impact of a three-month intervention involving ONS and physical rehabilitation on post-ICU COVID-19 patients. The findings suggest notable improvements in nutritional status, muscle strength, and functional capacity. After the functional and nutritional intervention, patients’ weight gain occurred primarily through increases in muscle mass, without significant changes in fat mass, as indicated by BIA and nutritional ultrasound. Additionally, the intervention was generally well tolerated, with good adherence rates, indicating that it may be feasible for this patient population.

During hospital discharge, screening, diagnosis, and treatment of malnutrition are essential for all patients with COVID-19, especially for those with longer ICU stays [11]. Upon discharge, post-ICU COVID-19 patients experience varying degrees of malnutrition, often accompanied by sudden and substantial weight loss during their hospital stay that influences the quality of their lives [4,28,29,30]. Functional decline is also common [31,32,33,34]. In addition, sarcopenia is another major complication that can occur alongside COVID-19, especially following an ICU stay, and is often neglected [11,14]. Sarcopenia is associated with an increased risk of mortality during hospitalization, as well as disabilities, falls, and functional limitations after discharge [9]. It may have long-term effects that can result in persistent functional disability one year after discharge. It can also be associated with a significantly higher risk of re-admission in old people [9,35,36]. Survivors of ARDS experience significant physical impairments of muscle strength, walking capacity, and physical activity levels (physical SF-36 score) between six months and two years after being discharged from the ICU [37]. In addition, sarcopenia in post-critical COVID-19 ambulatory patients is difficult to assess due to the limitations of techniques such as BIA or dual-energy X-ray absorptiometry (DXA) and the high prevalence of obesity in this population [14]. In this context, nutritional ultrasound is emerging as a useful technique for the evaluation of malnutrition and sarcopenia in patients with a normal or elevated BMI [4,14].

In our study, all patients were malnourished at baseline (hospital discharge) according to the SGA or GLIM criteria [4]. Other studies also reported a significant prevalence of malnutrition [28,29,30]. Overweight and obesity were highly prevalent in our cohort, which has been reported by other authors as well [38]. Over 60% of the patients showed dynapenia, with a handgrip strength below the values recommended by the EWGSOP2 (<27 kg for men and <16 kg for women), and nearly 30% had TUG test scores above the cutoff value of 20 s. The degree of physical dysfunction was consistent with findings from other similar studies [28,29,30]. In addition, according to the GLIM criteria [22], over 30% of men and women had reduced muscle mass as measured by FFMI (<17 for men and <15 kg/m^2^ for women), and over 40% had reduced muscle mass as measured by the ASMM index (<7 for men and <5.7 kg/m^2^ for women).

The ESPEN guidelines recommend offering COVID-19 patients hypercaloric (400 kcal/day) and hyperproteic (30 g/day) ONS after hospital discharge [11]. However, data about the nutritional and functional benefits of this approach in post-ICU COVID-19 patients are limited. A study of 38 critically ill COVID-19 patients showed that nutritional recovery seems to be very slow. The prevalence of malnutrition remained high (up to 66%) three months after discharge. Only a minority (10 patients) received nutritional support within 3 months of ICU discharge, suggesting that malnutrition was either underdiagnosed or undertreated in this population [5]. An observational longitudinal study described the evolution of nutritional parameters between admission and 30 days after hospital discharge in 91 patients admitted for COVID-19 who received early nutritional management (33% required admission to the ICU). During hospitalization, all patients received optimized nutritional management according to international and French guidelines on nutritional screening and support, but not physical rehabilitation. Thirty days after discharge, 28.6% of patients hospitalized for COVID-19 were malnourished, compared to 42.3% at admission [6]. Body composition was not examined. Lakenman et al. focused on a cohort of post-ICU COVID-19 patients, assessing their nutritional and physical recovery one year after ICU discharge. The results indicated that while most patients regained their body weight, 19% continued to have low FFMI and 50% had high fat mass, highlighting a persistent imbalance in body composition [7]. The intervention included dietary recommendations. In contrast, at hospital discharge and three months after, our patients were offered a serum lactoprotein enriched with leucine and vitamin D, along with simple and non-strenuous physical rehabilitation. Additionally, we thoroughly examined body composition.

Evidence shows a possible positive impact of physical activity in combination with supplementation of amino acids or their metabolites on muscle mass and strength [39,40]. Physical exercise or sufficient amounts of essential amino acids such as leucine can be anabolic triggers for protein synthesis [41]. Vitamin D plays a role in lowering the anabolic threshold for the anabolic stimulation of muscle protein synthesis by leucine [42]. Vitamin D deficiency negatively impacts muscle metabolism, which can be restored by vitamin D supplementation [43]. In addition, the absorption rate of dietary amino acids by the intestine influences the rate of postprandial protein synthesis, breakdown, and, ultimately, protein deposition. Serum lactoprotein intake results in a rapid, high, and transient increase in plasma amino acid levels [44], which has been associated with an increased rate of protein synthesis [45,46].

The intervention results revealed a significant improvement in nutritional status 90 days after hospital discharge. By the end of this period, 88% of the patients had a normal nutritional status by SGA and 63.5% by GLIM criteria. Regarding functionality, a significant improvement was achieved in parameters associated with patients’ fragilities, such as handgrip strength (significant improvement of approximately 50%) and the TUG test (42% improvement). Nutritional and functional improvements were of a similar magnitude in men and women and there were no significant problems of tolerance or adherence.

In terms of body composition by BIA, improvements were achieved in FFMI (with a 19.3% increase and improvements in 50% of patients with very low FFMI), SMMI (with a 19.1% increase), ASMM index (with an 11.4% increase), and PhA (with a 24.3% increase). In the multivariate study, patients with worse nutritional status, as determined by a lower baseline PhA, exhibited greater improvement in PhA after the intervention. Conversely, older patients demonstrated lower PhA improvement rates. This suggests that older individuals may require greater support and closer monitoring. However, the expected improvement will be significant even in those with worse baseline conditions. Given the association between low PhA and higher mortality in COVID-19 patients [10], improving this parameter emphasizes the necessity of the intervention.

It is important to clarify that the weight gain observed in our study seems largely attributable to muscle mass, as suggested by several objective measures. Notably, FFMI increased by 19.3%, SMMI rose by 19.1%, and ASMMI showed an 11.4% increase (Table 4). Additionally, FFM increased by 6.4 kg, accompanied by gains in body cell mass, which may indicate that muscle mass contributed significantly to the overall weight gain. Further supporting this, we observed improvements in functional capacity, with handgrip strength increasing by 48.2% over 90 days (Table 3), which likely reflects gains in muscle strength. Ultrasound measurements also demonstrated a 47.5% increase in RFCSA (Table 5) and notable improvements in other muscle parameters, such as circumference, *X*-axis, and *Y*-axis. RFCSA and *Y*-axis, in particular, showed the most significant gains in both men and women (Table S6).

It should also be mentioned that although the patients gained an average of 6.8 kg (an increase of 8.9%) after three months of intervention, there were no significant increases in either fat mass by BIA or in total, abdominal, or preperitoneal adipose tissue, or in rectus femoris subcutaneous adipose tissue by nutritional ultrasound. Although the obesity rate was high in our study, the intervention is unlikely to be associated with increased cardiovascular risk because the weight gain was primarily due to muscle mass rather than fat mass.

It should also be noted that improvements in parameters such as PhA, RFCSA, *X*-axis, or *Y*-axis were associated with improvements in handgrip strength and, in some variables, with improvements in the TUG test. Therefore, the improvements in parameters indicating sarcopenia were statistically significant and might have evident clinical applications.

Physical rehabilitation plays a crucial role in improving physical function and recovery for post-ICU patients, including those recovering from severe COVID-19. Studies have demonstrated significant improvements in exercise capacity, muscle strength, and balance following tailored rehabilitation programs, with outcomes measured by the 6 min walk test, sit-to-stand test, and gait speed assessments [47,48]. These interventions not only restore physical function but also address complications like muscle loss and respiratory impairment, making rehabilitation a vital component of post-ICU care [47,48].

Our study had some limitations that ought to be taken into consideration. We acknowledge that lacking a control group, this exploratory study has inherent limitations in establishing causality between the intervention and the observed outcomes. Since this was an uncontrolled study, we cannot exclude the possibility that part or all of the observed improvements in nutritional and functional status may be attributable to the natural recovery process after critical illness, rather than to the intervention itself. However, our preliminary findings are valuable in guiding future research and randomized controlled trials to confirm the efficacy of this intervention in a broader population. Another limitation of this study is the sample size and the number of participants lost during follow-up. Although the sample size of 96 patients is significant, it might not fully represent all COVID-19 patients who have undergone ICU stays. The final sample size was slightly smaller compared to the initial planned number. Although a formal power analysis was conducted, and we anticipated a 20% loss to follow-up, only 85 patients completed the study, slightly lower than the projected 96 patients. This reduction in sample size may limit the generalizability of our findings. Future studies with larger sample sizes are necessary to validate these findings further and improve the ability to generalize the results to broader populations. In addition, some measurements, such as waist circumference, can be operator-dependent despite following a standardized protocol, which may introduce variability and affect the reproducibility of the results. Moreover, while the dropout rate was relatively low, it is important to acknowledge that the outcomes of these patients were not tracked. However, in most cases, follow-up would not have been feasible due to the nature of the reasons for dropout, which included loss to follow-up, death, or the patient’s decision to withdraw from the study. Additionally, adherence to the intervention was likely compromised among those who dropped out, which may have limited the utility of following up with them. Future studies should minimize loss to follow-up and, where feasible, explore strategies to assess the outcomes of patients who do not complete the study. The number of patients who declined to participate was not systematically recorded, which may have also introduced a selection bias.

In addition, this research was conducted during the fourth and sixth outbreaks of COVID-19 in Spain [49]. The findings may not fully apply to other settings or to different or future variants of SARS-CoV-2. Patients with obesity may have their muscle mass overestimated, which could lead to an underdiagnosis of malnutrition when the GLIM criteria are used. However, our study’s strength is that we ensured consistency in the nutritional ultrasound results by providing specific training to the researchers involved. Additionally, the same US models and protocols were utilized in all hospitals participating in the study, and all investigators received training to adhere to a specific nutritional ultrasound protocol before commencing the study to minimize any potential variability in the results. Nevertheless, observer intra- and inter-observer variability remains an inherent limitation of ultrasound-based body composition assessment [16] and should be considered when interpreting our results. Finally, the study lacks a cost-effectiveness analysis. While the study primarily focused on clinical and nutritional outcomes, the potential resource requirements of the intervention were not evaluated. Future research should include an economic analysis to better understand the feasibility and financial implications of implementing this intervention on a larger scale.

## 5. Conclusions

In conclusion, our study emphasizes the importance of conducting a comprehensive nutritional assessment for post-ICU COVID-19 patients, focusing on nutrient balance, body composition, functional status, and biochemical status. A three-month intervention including oral nutritional supplements of 100% serum lactoprotein enriched with leucine and vitamin D and physical rehabilitation was associated with significant nutritional and functional status improvement. Patients gained weight primarily by increasing their muscle mass. As measured by BIA, there was no significant increase in fat mass or in total, superficial, or preperitoneal abdominal fat, as evaluated by nutritional ultrasound. The intervention was well tolerated, and adherence was good. However, due to the study’s observational nature and the absence of a control group, these results should be interpreted with caution. While the findings are clinically meaningful, they are exploratory, and future randomized controlled trials are needed to confirm the efficacy of this intervention in broader populations. A long-term study will also be necessary to observe the evolution of these results and further validate the positive impact of integrated nutritional and physical rehabilitation strategies in the recovery of post-critical COVID-19 patients.

## Figures and Tables

**Table 1:** Demographic, clinical, and functional baseline characteristics of the population.

	Total	Men	Women	*p*-Value
*n* (%)	96 (100.0)	69 (71.9)	27 (28.1)	-
Age, mean (SD), years	58.8 (8.5)	58.5 (8.8)	59.7 (7.7)	NS
Age ≥ 65 years, *n* (%)	29 (30.2)	20 (29.0)	9 (33.3)	NS
Co-morbidities, *n* (%)				
Obesity	40 (41.7)	24 (34.8)	16 (59.3)	0.0386 *
HBP	34 (35.4)	22 (31.9)	12 (44.4)	NS
Diabetes mellitus	19 (19.8)	13 (18.8)	6 (22.2)	NS
COPD	6 (6.3)	5 (7.2)	1 (3.7)	NS
CKD	2 (2.1)	2 (2.9)	0 (0.0)	NS
CHF	3 (3.1)	3 (4.3)	0 (0.0)	NS
Active oncologic pathology	2 (2.1)	2 (2.9)	0 (0.0)	NS
Length of hospital stay, mean (SD), days	48.2 (37.6)	49.7 (40.9)	44.6 (27.6)	NS
Pre-ICU hospital stay, mean (SD), days	2.3 (3.2)	2.0 (2.4)	3.1 (4.7)	NS
ICU stay, mean (SD), days	28.7 (7.5)	30.3 (29.7)	24.4 (20.4)	NS
SOFA score, mean (SD)	4.1 (2.4)	4.2 (2.7)	4.0 (2.3)	NS
Mechanical ventilation, *n* (%)	58 (60.4)	40 (58.0)	18 (66.7)	NS
NIMV	5 (5.2)	4 (5.8)	1 (3.7)	NS
HFNC	33 (34.4)	25 (36.2)	8 (29.6)	NS
CRP, median (IQR), mg/dL	2.9 (0.5–9.0)	2.9 (0.4–11.3)	2.9 (0.7–8.4)	NS
Vitamin D, median (IQR), mg/dL	13.1 (10–17)	15.5 (9.8–18)	11.7 (10–13.1)	NS
BI score at hospital discharge, median (IQR)	90 (65–100)	90 (65–100)	85 (70–95)	NS
BI < 100, *n* (%)	64 (66.7)	42 (60.9)	22 (81.5)	NS
Handgrip strength				
Mean (SD), kg	21.6 (11.0)	25.0 (10.9)	13.0 (4.9)	<0.0001
<27 men and <16 women; *n* (%)	60 (62.5)	39 (56.5)	21 (77.8)	NS
TUG test				
Mean (SD), seconds	19.9 (17.2)	16.7 (14.2)	28.0 (21.5)	0.0004
>20 s, *n* (%)	26 (27.1)	14 (20.3)	12 (44.4)	0.0224

Abbreviations: BI, Barthel index; BMI, body mass index; CHF, congestive heart failure; CKD, chronic kidney disease; COPD, chronic obstructive pulmonary disease; CRP, C-reactive protein; HBP, high blood pressure; HFNC, high-flow nasal cannula; ICU, intensive care unit; IQR, interquartile range; NIMV, non-invasive motion ventilation; NS, not statistically significant; SD, standard deviation; SOFA, Sequential Organ Failure Assessment; TUG, Timed Up and Go test. * *p*-value: comparison between BMI < 30 kg/m^2^ and ≥30 kg/m^2^ (Fisher’s exact test).

**Table 2:** Changes in clinical nutritional status after 90 days of intervention.

	Day 0 *	Day 90 **	*p*-Value	Change	
				Δ	%
Weight, mean (SD), kg	81.0 (16.8)	87.8 (17.5)	<0.00001	6.8 (5.2)	8.9% (6.6)
BMI, mean (SD), kg/m^2^	28.6 (6.0)	30.7 (6.7)	<0.00001	1.8 (4.8)	7.6% (15.0)
Waist circumference, mean (SD), cm	103.7 (15.0)	107.5 (14.3)	<0.00001	4.0 (7.5)	4.3% (6.9)
Malnutrition by SGA, *n* (%)					
SGA A	0 (0)	75 (88.2)	<0.00001		88.2%
SGA B	50 (52.1)	10 (11.8)			−40.3%
SGA C	46 (47.9)	0 (0)			−47.9%
Malnutrition by GLIM criteria, *n* (%)					
No malnutrition	0 (0)	54 (63.5)	<0.00001		63.5%
Moderate	44 (45.8)	24 (28.2)			−17.6%
Severe	52 (54.2)	7 (8.2)			−46.0%

Abbreviations: BMI, body mass index, GLIM, Global Leadership Initiative on Malnutrition; SD, standard deviation; SGA, Subjective Global Assessment. * The number of patients at day 0 was 96, with 69 males and 27 females. ** The number of patients at day 90 was 85, with 62 males and 23 females.

**Table 3:** Changes in functional status (Barthel index, handgrip strength, and TUG test) after 90 days of intervention.

	Day 0 *	Day 90 **	*p*-Value	Change	
				Δ	%
BI < 100, *n* (%)	64 (66.7)	23 (27.0)	<0.0001		−39.7%
Handgrip strength					
Mean (SD), kg	21.6 (11.0)	28.9 (11.7)	<0.00001	7.3 (6.7)	48.2% (55.6)
<27 men and <16 women; *n* (%)	60 (62.5)	26 (31.3)	<0.0001		−31.2%
TUG test					
Mean (SD), seconds	19.9 (17.2)	8.9 (4.2)	<0.0001	−10.5 (15.5)	−41.4% (24.7)
> 20 s, *n* (%)	26 (27.1)	4 (4.7)	<0.0001		−22.4%

Abbreviations: BI, Barthel index; SD, standard deviation; TUG, Timed Up and Go test. * The number of patients at day 0 was 96, with 69 males and 27 females. ** The number of patients at day 90 for IB and TUG was 85 (62 males and 23 females), and for handgrip strength was 83 (61 males and 22 females).

**Table 4:** Changes in bioelectrical impedance analysis after 90 days of intervention.

	Day 0		Day 90		*p*-Value	Change	
	*n*	Result	*n*	Result		Δ	%
Weight, mean (SD), kg	92	80.8 (16.7)	83	87.4 (17.5)	<0.00001	6.6 (4.8)	8.8% (6.4)
Fat mass, mean (SD), kg	92	28.9 (11.6)	83	29.7 (11.3)	0.281	0.4 (6.9)	5.5% (25.4)
Fat-free mass, mean (SD), kg	90	49.6 (13.3)	76	55.2 (14.5)	<0.00001	6.4 (9.5)	19.7% (55.7)
FFMI, mean (SD), kg/m^2^	90	17.3 (3.9)	76	19.3 (4.2)	<0.00001	2.2 (3.4)	19.6% (55.0)
FFMI < 17 men and <15 kg/m^2^ women; *n* (%)	90	30 (33.3)	76	13 (17.1)	0.0087		−16.2%
Body cell mass, mean (SD), kg	78	28.2 (7.6)	63	34.4 (8.7)	<0.00001	5.2 (4.4)	21.1% (20.75)
SMMI, mean (SD), kg/m^2^	67	8.5 (2.4)	59	9.6 (3.8)	<0.00001	1.4 (3.5)	19.1% (48.5)
Total body water, mean (SD), L	86	39.3 (8.4)	79	43.4 (9.0)	<0.00001	4.3 (4.4)	11.5% (11.0)
ASMM index, mean (SD), kg/m^2^	49	7.2 (2.1)	41	7.7 (1.8)	0.0002	0.6 (1.8)	11.4% (20.0)
ASMM index < 7 men and <5.7 kg/m^2^ women, *n* (%)	49	20 (40.8)	41	12 (29.2)	0.127		−11.6%
PhA, mean (SD), degrees	87	4.5 (1.0)	81	5.4 (0.9)	<0.00001	0.9 (0.7)	24.3% (22.2)
PhA < 3.95°, *n* (%)	87	26 (29.8)	81	3 (3.7)	<0.0001		−26.1%
Standardized PhA, median (IQR)	31	−2.0 (−2.7 to −1.0)	28	−1.2 (−1.8 to −0.7)	0.0071	0.5 (−0.2 to 1.7)	34.8% (−11 to 66)
RZ, mean (SD), ohmios	55	529.3 (108.0)	49	465.2 (90.3)	<0.00001	−74.3 (77.7)	−12.8% (13.7)
XC, mean (SD), ohmios	55	43.1 (11.3)	49	44.6 (9.2)	0.267	1.3 (8.0)	5.5% (19.5)

Abbreviations: ASMM, appendicular skeletal muscle mass; FFMI, fat-free mass index; IQR, interquartile range, PhA, phase angle; RZ: raw resistance; SMMI, skeletal muscle mass index; XC: reactance.

**Table 5:** Changes in nutritional ultrasound assessments after 90 days of intervention.

	Day 0		Day 90			Change	
	*n*	Result	*n*	Result	*p*-Value	Δ	%
Rectus femoris							
RFCSA, mean (SD), cm^2^	96	3.3 (1.3)	85	4.6 (1.7)	<0.00001	1.2 (1.4)	47.5% (61.0)
Muscle circumference, mean (SD), cm	96	8.7 (1.3)	85	9.3 (1.5)	<0.0001	0.57 (1.6)	8.2% (20.8)
*X*-axis, mean (SD), cm	96	3.6 (0.6)	85	3.8 (0.6)	0.0260	0.15 (0.7)	7.8% (32.7)
*Y*-axis, mean (SD), cm	96	1.0 (0.3)	85	1.3 (0.4)	<0.00001	0.35 (0.3)	41.4% (43.3)
Subcutaneous adipose tissue (SD), cm	96	0.9 (0.5)	85	0.9 (0.5)	0.570	−0.0004 (0.2)	7.1% (31)
Abdominal wall							
Total adipose tissue, mean (SD), cm	95	2.1 (1.0)	84	2.0 (0.9)	0.207	−0.105 (0.6)	−0.6% (27.3)
Superficial adipose tissue, mean (SD), cm	95	1.0 (0.6)	84	0.9 (0.5)	0.338	−0.05 (0.4)	5.1% (49.4)
Preperitoneal adipose tissue mean (SD), cm	95	0.8 (0.4)	84	0.8 (0.5)	0.799	−0.005 (0.5)	15.2% (101)

Abbreviations: RFCSA, rectus femoris cross-sectional area; X-axis: longitudinal axis; Y-axis; transversal axis; SD: standard deviation.

**Table 6:** Correlations between baseline variables and percentage changes in phase angle (univariate analysis).

Baseline Characteristics	Beta	95%CI	*p*
Male/female	−9.1026	−20.9 to 2.7	0.130
Age, years	−0.640	−1.216 to −0.064	0.030
Diabetes, yes/no	−16.942	−33.097 to −0.787	0.040
Length of hospital stay, days	0.237	0.117 to 0.357	<0.0001
ICU stay, days	0.398	0.239 to 0.556	<0.0001
SOFA score	2.461	−3.513 to 8.435	0.387
CRP, mg/dL	−0.011	−0.068 to 0.045	0.690
Vitamin D, mg/dL	−0.209	−1.403 to 0.983	0.720
BI score	−0.607	−1.539 to 0.325	0.199
Handgrip strength, kg	−0.666	−1.115 to −0.218	0.004
TUG, seconds	0.682	0.366 to 0.998	<0.0001
SMMI, kg/m^2^	−1.444	−3.893 to 1.003	0.242
PhA, grades	−13.495	−17.242 to −9.749	<0.0001

Abbreviations: BI, Barthel index; CI, confidence interval; CRP, C-reactive protein; ICU, intensive care unit; SMMI, skeletal muscle mass index; SOFA, Sequential Organ Failure Assessment; PhA, phase angle; TUG, Timed Up and Go test.

## Data Availability

Data supporting the findings of this study are available from the corresponding author on reasonable request.

## Supplementary Materials

The following supporting information can be downloaded at https://www.mdpi.com/article/10.3390/nu17101722/s1. NutriEcoMuscle Study Team. Table S1: Oral supplement nutritional information and ingredients (Fortimel^®^ Advanced). Table S2. Study assessments. Table S3. Changes in clinical nutritional status after 90 days of intervention stratified by sex. Table S4. Changes in functional status (Barthel index, handgrip strength, and TUG test) after 90 days of intervention stratified by sex. Table S5. Changes in bioelectrical impedance analysis after 90 days of intervention stratified by sex. Table S6. Correlations between the percentage change after the intervention of variables measured in BIA and ultrasound. Table S7. Correlations between the percentage change after the intervention in BIA and ultrasound measurements and the percentage change in handgrip strength and TUG results. Table S8. Tolerance after 45 and 90 days of the intervention.

## Abbreviations

The following abbreviations are used in this manuscript:

ARDS	Acute Respiratory Distress Syndrome
ASMM	Appendicular skeletal muscle mass
ASMMI	Appendicular skeletal muscle mass index
BI	Barthel index
BIA	Bioelectrical impedance analysis
BIVA	Bioelectrical impedance vector analysis
BMI	Body mass index
CHF	Congestive heart failure
CI	Confidence interval
CKD	Chronic kidney disease
COPD	Chronic obstructive pulmonary disease
COVID-19	Coronavirus disease 2019
CRP	C-reactive protein
DM	Diabetes mellitus
DXA	Dual-energy X-ray absorptiometry
ESPEN	European Society for Clinical Nutrition and Metabolism
EWGSOP2	European Working Group on Sarcopenia in Older People 2
FFM	Fat-free mass
FFMI	Fat-free mass index
GLIM	Global Leadership Initiative on Malnutrition criteria
HBP	High blood pressure
HFNC	High flow nasal cannula
ICU	Intensive care unit
IQR	Interquartile range
NIMV	Non-invasive mechanical ventilation
NS	Not statistically significant
ONS	Oral nutritional supplement
PhA	Phase angle
RFCSA	Rectus femoris cross-sectional area
RT-PCR	Reverse Transcription Polymerase Chain Reaction
SD	Standard deviation
SGA	Subjective Global Assessment
SMMI	Skeletal muscle mass index
SOFA	Sequential Organ Failure Assessment Score
SPA	Standardized phase angle
TUG	Timed Up and Go test
