# Supplementary material for: Nutritional and Physical Rehabilitation in Post-Critical Coronavirus Disease 2019 (COVID-19) Ambulatory Patients: The NutriEcoMuscle Study
Source: Nutrients. 2025 May 20;17(10):1722. doi: 10.3390/nu17101722 (PMC12113686; doi:10.3390/nu17101722)
Supplement: Supplementary file 1 [file nutrients-17-01722-s001.zip › nutrients-3611288-supplementary.pdf]

Supplementary material

EcoNutriMuscle Study team (in alphabetical order) 2

Table S1. Oral supplement nutritional information and ingredients (Fortimel® Advanced)[1]. 7

Table S2. Study assessments. 9

Table S3. Changes in clinical nutritional status after 90 days of intervention stratified by sex. 10

Table S4. Changes in functional status (Barthel index, hand-grip strength, and TUG test) after 90 days of intervention stratified by sex...................................11

Table S5. Changes in bioelectrical impedance analysis after 90 days of intervention stratified by sex. 12

Table S6. Correlations between the percentage change after the intervention of variables measured in BIA and ultrasound..14

Table S7. Correlations between the percentage change after the intervention in BIA and ultrasound measurements and the percentage change in handgrip strength and TUG results. 16

Table S8. Tolerance after 45 and 90 days of the intervention.............................18

EcoNutriMuscle Study team (in alphabetical order)

Name	Department	Hospital	Address
**Principal Investigators**			
**Bellido Guerrero, D**	Department of Endocrinology and Nutrition	Complejo Hospitalario Universitario de Ferrol (A Coruña)	Av. da Residencia, S/N, 15405 Ferrol, A Coruña, Spain;
**Botella-Romero, F**	Spanish Society of Endocrinology and Nutrition	Sociedad Española de Endocrinología y Nutrición	C/Villalar, 7, 28001, Madrid, Spain;
**Bretón-Lesmes, I**	Department of Endocrinology and Nutrition	Hospital General Universitario Gregorio Marañón	C/Dr. Esquerdo, 46, 28007, Madrid, Spain
**García-Almeida, JM**	Department of Endocrinology and Nutrition	Hospital Virgen de la Victoria	Campus de Teatinos,S/N,Puerto de la Torre, Málaga, Spain
**Joaquín-Ortiz, C**	Department of Endocrinology and Nutrition	Hospital Germans Trias i Pujol	08916, Badalona, Spain.
**Martínez-Olmos, MA**	Department of Endocrinology and Nutrition	Hospital General Universitario de Santiago	Rúa da Choupana, S/N, 15706 Santiago de Compostela, A Coruña, Spain
**Matía-Martín, P**	Department of Endocrinology and Nutrition	Hospital Clínico Universitario San Carlos	C/Prof Martín Lagos, S/N, 28040, Madrid, Spain
**Ocón-Bretón, MJ**	Department of Endocrinology and Nutrition	Hospital Clínico Universitario Lozano Blesa	C/San Juan Bosco, 15, 50009, Zaragoza, Spain

**Riestra-Fernández, M**	Department of Endocrinology and Nutrition	Hospital Universitario de Cabueñes	Los Prados, 395, 33394, Gijón, Asturias, Spain
**Zabalegui, A**	Nutritional Support Unit	Hospital Universitario Vall d'Hebron	Pg. de la Vall d'Hebron, 119 Horta-Guinardó, 08035, Barcelona, Spain
**Zugasti-Murillo, A**	Section of Clinical Nutrition and Dietetics	Complejo Hospitalario Universitario de Navarra	C/de Irunlarrea, 3, 31008, Pamplona, Navarra, Spain
**Co-investigators**			
**Burgos-Peláez, R**	Nutritional Support Unit	Hospital Universitario Vall d'Hebron	Pg. de la Vall d'Hebron, 119 Horta-Guinardó, 08035, Barcelona, Spain
**Cárdenas, G**	Nutritional Support Unit	Hospital Universitario Vall d'Hebron	Pg. de la Vall d'Hebron, 119 Horta-Guinardó, 08035, Barcelona, Spain
**Diaz-Naya, L**	Department of Endocrinology and Nutrition	Hospital Universitario de Cabueñes	Los Prados, 395, 33394, Gijón, Asturias, Spain
**Escuer-Núñez, I**	Section of Clinical Nutrition and Dietetics	Hospitalario Universitario de Navarra	C/de Irunlarrea, 3, 31008, Pamplona, Navarra, Spain
**Estany Quera C**	Fundació lluita contra les infeccions	Hospital Universitari Germans Trias i Pujol	Carretera del canyet S/N, 08916, Badalona, Spain.
**Fernández-Pombo A**	Division of Endocrinology and Nutrition	University Clinical Hospital of Santiago de Compostela	Rua da Choupana S/N, 15706, Santiago de Compostela, Spain
**Gutioerrez-Buey, G**	Department of Endocrinology and Nutrition	Hospital Universitario de Cabueñes	Los Prados, 395, 33394, Gijón, Asturias, Spain
**Hernández-Núñez MG**	Department of Endocrinology and Nutrition	Hospital Clínico San Carlos, Instituto de	C/Prof Martín Lagos, S/N, 28040, Madrid, Spain

		Investigación Sanitaria San Carlos (IdISSC)	
**Larrad-Sáinz, A**	Department of Endocrinology and Nutrition	Hospital Clínico San Carlos, Instituto de Investigación Sanitaria San Carlos (IdISSC)	C/Prof Martín Lagos, S/N, 28040, Madrid, Spain
**Maichle, S**	Critical Unit	Hospital Clínico San Carlos, Instituto de Investigación Sanitaria San Carlos (IdISSC)	C/Prof Martín Lagos, S/N, 28040, Madrid, Spain
**Marcuello-Foncillas, C**	Department of Endocrinology and Nutrition	Hospital Clínico San Carlos, Instituto de Investigación Sanitaria San Carlos (IdISSC)	C/Prof Martín Lagos, S/N, 28040, Madrid, Spain
**Palmas Cadía, F**	Nutritional Support Unit	Hospital Universitario Vall d'Hebron	Pg. de la Vall d'Hebron, 119 Horta-Guinardó, 08035, Barcelona, Spain
**Pazos-Guerra, M**	Department of Endocrinology and Nutrition	Hospital Clínico San Carlos, Instituto de Investigación Sanitaria San Carlos (IdISSC)	C/Prof Martín Lagos, S/N, 28040, Madrid, Spain
**Pena Dubra, A**	Division of Endocrinology and Nutrition	University Clinical Hospital of Ferrol (A Coruña)	Ctra. de la Residencia, S/N, 15405, Ferrol, Spain
**Pérez-Ferre, N**	Department of Endocrinology and Nutrition	Hospital Clínico San Carlos, Instituto de	C/Prof Martín Lagos, S/N, 28040, Madrid, Spain

		Investigación Sanitaria San Carlos (IdISSC)	
**Ramos Rodas, A**	Department of Endocrinology and Nutrition	Hospital Universitari Germans Trias i Pujol	Carretera del canyet S/N, 08916, Badalona, Spain.
**Rendón-Barragán, H**	Section of Clinical Nutrition and Dietetics	Hospitalario Universitario de Navarra	C/de Irunlarrea, 3, 31008, Pamplona, Navarra, Spain
**Rodriguez-Carnero MG**	Division of Endocrinology and Nutrition	University Clinical Hospital of Santiago de Compostela	Rua da Choupana S/N, 15706, Santiago de Compostela, Spain
**Román Eyo, AP**	Division of Endocrinology and Nutrition	University Clinical Hospital of Santiago de Compostela	Rua da Choupana S/N, 15706, Santiago de Compostela, Spain
**Ros Martí, M**	Department of Endocrinology and Nutrition	Hospital Universitari Germans Trias i Pujol	Carretera del canyet S/N, 08916, Badalona, Spain.
**Sallán-Díaz, L.**	Section of Clinical Nutrition and Dietetics	Hospital Clínico Universitario Lozano Blesa	C/San Juan Bosco, 15, 50009 Zaragoza, Spain
**Sanchez Bao, AM**	Department of Endocrinology and Nutrition	Complejo Hospitalario Universitario de Ferrol (A Coruña)	Av. da Residencia, S/N, 15405, Ferrol, A Coruña, Spain.
**Sospedra Martínez, M**	Department of Endocrinology and Nutrition	Hospital Universitari Germans Trias i Pujol	Carretera del canyet S/N, 08916, Badalona, Spain.
**Urdaniz Borque, R**	Department of Endocrinology and Nutrition	Hospital Clínico Universitario Lozano Blesa	C/San Juan Bosco, 15, 50009, Zaragoza, Spain

Table S1. Oral supplement nutritional information and ingredients (Fortimel® Advanced)[1].

Nutritional Information	Per 100ml
**Energy:**	151kcal (633kJ)
**Fat:**	5.2g
of which	
- Saturates	0.46g
**Carbohydrate:**	15.0g
of which	
- Sugars	8.1g
- Lactose	<0.2g
**Fibre**	1.4g
**Protein**	10.4g
**Salt:**	0.17g
**Vitamins:**	
Vit. A	75.0μg
Vit. D	5.00μg
Vit. E	2.40mg (α-TE/ET)
Vit. K	9.00μg
Thiamin	0.13mg
Riboflavin	0.16mg
Niacin	0.45mg
Pantothenic acid	0.50mg
Vit. B_6	0.38mg
Folic acid	40.0μg
Vit. B_{12}	0.58μg
Biotin	3.00μg
Vit. C	16.0mg
**Minerals and trace elements:**	
Na	73mg
K	160mg
Cl	124mg
Ca	200mg
P	100mg

Mg	30.3mg
Fe	1.25mg
Zn	1.12mg
Cu	0.14mg
Mn	0.26mg
F	0.06mg
Mo	7.23µg
Se	9.17µg
Cr	4.43µg
l	13.5µg
**Others:**	
Choline	40.0mg
L-Leucine	1.5g
Osmolarity	680mOsmol/l

Ingredients

Tropical Vanilla Flavor

Ingredients: Water, glucose syrup, whey protein (from cow's milk), vegetable oils (rapeseed oil, high oleic sunflower oil, sunflower oil), sucrose, dietary fibres (Galacto-oligosaccharides (from cow's **milk**), pectin, oligofructose, inulin)), calcium citrate, acidity regulator (lactic acid), L-leucine, flavour (vanilla), potassium citrate, magnesium chloride, emulsifier (soy **lecithin**), sodium citrate, L-valine, L-isoleucine, choline chloride, L-ascorbic acid, maltodextrin, sweeteners (acesulfame-K, sucralose), ferrous lactate, DL-α-tocopheryl acetate, zinc sulphate, colour (curcumin), nicotinamide, copper gluconate, manganese sulphate, calcium D-pantothenate, pyridoxine hydrochloride, thiamin hydrochloride, riboflavin, retinyl acetate, sodium fluoride, pteroylmonoglutamic acid, chromium chloride, sodium molybdate, potassium iodide, sodium selenite, cholecalciferol, phytomenadione, D-biotin, cyanocobalamin

SOURCE:

1. Danone. Fortimel Advanced with ActiSyn™. [cited 2024 May 20]; Available from: https://www.nutricia.com/products/frailty-and-drm/fortimel-advanced.html

Table S2. Study assessments.

	Baseline	Intermediate Visit	Month 3
	In-person visit	Telephone visit	In-person visit
Time	Hospital discharge (day 0)	Day 45±5	Day 90±5
Demographic data	X		
Medical history	X		
Hospital stay information	X		
Nutritional status	X		X
Nutritional requirements*	X		X
Barthel index	X		X
Anthropometric data	X		X
GLIM criteria	X		X
Handgrip strength	X		X
TUG test	X		X
Nutritional ultrasound	X		X
Bioelectrical impedance	X		X
Quality of life**	X		X
Analytics	X		X
ONS tolerance		X***	X
Adherence to ONS		X***	X

Abbreviations: GLIM, Global Leadership Initiative on Malnutrition; ONS, oral nutritional supplement; TUG, Timed Up and Go.

* Percentage of nutritional requirements the patient met with the conventional diet: 25%, 50%, 75%, 100%.

** The results of the quality of life assessments will be presented in a separate article.

*** All patients completed a diary during the study to collect information related to adherence and tolerance to treatment. Unused vials were counted on a daily basis. In addition, patients were be asked to complete a gastrointestinal tolerability questionnaire. This data was recorded by the investigators at the interim telephone visit and at the 6-month face-to-face visit.

Table S3. Changes in clinical nutritional status after 90 days of intervention stratified by sex.

	Total					Males					Females				
	Day 0*	Day 90**	p-value	Change		Day 0	Day 90	p-value	Change		Day 0	Day 90	p-value	Change	
				Δ	%				Δ	%				Δ	%
Weight, mean (SD), kg	81.0 (16.8)	87.8 (17.5)	<0.00001	6.8 (5.2)	8.9% (6.6)	83.1 (16.9)	90,2 (17.5)	<0.00001	7.4 (5.2)	9.5% (6.9)	75.6 (15.3)	81.2 (16.3)	<0.0001	5.2 (4.4)	7.1% (5.6)
BMI, mean (SD), kg/m^2	28.6 (6.0)	30.7 (6.7)	<0.00001	1.8 (4.8)	7.6% (15.0)	27.7 (5.3)	30.2 (5,1)	<0.00001	2.4 (2.2)	9.8% (10.3)	31.1 (7.2)	31.9 (9.7)	0.0048	0.14 (8.3)	1.8% (22.5)
Waist circumference, mean (SD), cm	103.7 (15.0)	107.5 (14.3)	<0.00001	4.0 (7.5)	4.3% (6.9)	103.6 (13.9)	108.4 (13.9)	<0.00001	5.5 (6.0)	5.6% (6.1)	104.2 (17.9)	105.1 (15.5)	0.440	0.18 (9.7)	0.9% (7.7)
**Malnutrition by SGA, n (%)**															
SGA A	0 (0)	75 (88.2)			88.2%	0 (0)	56 (90.3)			90.3%	0 (0)	19 (82.6)			82.6%
SGA B	50 (52.1)	10 (11.8)	<0.00001		-40.3%	34 (49.2)	6 (9.7)	<0.00001		-39.5%	16 (59.2)	4 (17.4)	<0.00001		-41.8%
SGA C	46 (47.9)	0 (0)			-47.9%	35 (50.7)	0 (0)			-50.7%	11 (40.7)	0 (0)			-40.7%
**Malnutrition by GLIM criteria, n (%):**															
No malnutrition	0 (0)	54 (63.5)			63.5%	0 (0)	40 (64.5)			64.5%	0 (0)	14 (60.8)			60.8%
Moderate	44 (45.8)	24 (28.2)			-17,6%	30 (43.5)	20 (32.2)			-11.3%	14 (51.8)	4 (17.3)			-34.5%
Severe	52 (54.2)	7 (8.2)	<0.00001		-46.0%	39 (56.5)	2 (3.2)	<0.00001		-53.3%	13 (48.2)	5 (21.7)	<0.00001		-26.5%

Abbreviations: BMI, body mass index; GLIM, Global Leadership Initiative on Malnutrition; SD, standard deviation; SGA, Subjective Global Assessment.

*The number of patients at day 0 was 96, with 69 males and 27 females.

** The number of patients at day 90 was 85, with 62 males and 23 females.

Table S4. Changes in functional status (Barthel index, hand-grip strength, and TUG test) after 90 days of intervention stratified by sex.

	Total					Males					Females				
				Change					Change					Change	
	**Day 0***	**Day 90****	**p-value**	**Δ**	**%**	**Day 0**	**Day 90**	**p-value**	**Δ**	**%**	**Day 0**	**Day 90**	**p-value**	**Δ**	**%**
BI < 100, n (%)	64 (66.7)	23 (27.0)	<0.0001		-39.7%	42 (60.9)	10 (16.1)	<0.0001		-44.8%	22 (81.5)	13 (56.5)	0.0275		-25.0%
**Handgrip strength:**															
Mean (SD), kg	21.6 (11.0)	28.9 (11.7)	<0.00001	7.3 (6.7)	48.2% (55.6)	25.0 (10.9)	33.0 (10.6)	<0.00001	8.3 (7.0)	50.4% (58.6)	13.0 (4.9)	17.3 (5.5)	<0.0003	4.3 (4.6)	42.1% (47.0)
<27 men or <16 women; n (%)	60 (62.5)	26 (31.3)	<0.0001		-31.2%	39 (56.5)	18 (29.5)	0.001		-27.0%	21 (77.8)	8 (36.3)	0.0017		-41.5%
**TUG test:**															
Mean (SD), seconds	19.9 (17.2)	8.9 (4.2)	<0.0001	-10.5 (15.5)	-41.4% (24.7)	16.7 (14.2)	7.9 (3.7)	<0.0001	-8.48 (12.8)	-40.3% (23.8)	28.0 (21.5)	11.7 (4.1)	<0.0001	-16.0 (20.3)	-44.4% (27.2)
> 20 seconds, n (%)	26 (27.1)	4 (4.7)	<0.0001		-22.4%	14 (20.3)	2 (3.2)	0.0024		-17.1%	12 (44.4)	2 (8,6)	0.0052		-35.8%

Abbreviations: BI, Barthel index; SD, standard deviation; TUG, Timed Up and Go test.

*The number of patients at day 0 was 96, with 69 males and 27 females.

**The number of patients at day 90 for IB and TUG was 85 (62 males and 23 females), and for hand-grip strength was 83 (61 males and 22 females).

Table S5. Changes in bioelectrical impedance analysis after 90 days of intervention stratified by sex.

	Total							Males							Females						
	Day 0		Day 90		p-value	Change		Day 0		Day 90		p-value	Change		Day 0		Day 90		p-value	Change	
	n	Result	n	Result		Δ	%	n	Result	n	Result		Δ	%	n	Result	n	Result		Δ	%
Weight, mean (SD), kg	92	80.8 (16.7)	83	87.4 (17.5)	<0.00001	6.6 (4.8)	8.8% (6.4)	68	82.9 (17.0)	62	90.2 (17.5)	<0.00001	7.1 (4.8)	9.2% (6.6)	24	74.8 (14.6)	21	79.1 (15.1)	0.0002	5.1 (4.6)	7.4% (5.9)
Fat mass, mean (SD), kg	92	28.9 (11.6)	83	29.7 (11.3)	0.281	0.4 (6.9)	5.5% (25.4)	68	27.4 (11.6)	62	28.2 (10.8)	0.519	0.1 (7.4)	5.7% (28.0)	24	33.3 (10.7)	21	34.3 (12.0)	0.307	1.3 (5.2)	5.0% (15.3)
Fat-free mass, mean (SD), kg	90	49.6 (13.3)	76	55.2 (14.5)	<0.00001	6.4 (9.5)	19.7% (55.7)	67	53.0 (13.1)	56	60.0 (13.0)	<0.00001	7.7 (10.2)	23.9% (63.4)	23	39.7 (7.8)	20	41.6 (9.2)	0.08	2.5 (5.9)	7.0% (14.4)
FFMI, mean (SD), kg/m²	90	17.3 (3.9)	76	19.3 (4.2)	<0.00001	2.2 (3.4)	19.6% (55.0)	67	17.7 (4.0)	56	20.1 (4.0)	<0.00001	2.6 (3.6)	23.8% (62.6)	23	16.3 (3.3)	20	17.1 (4.2)	0.0023	1.1 (2.4)	7.0% (14.4)
FFMI <17 men or <15 kg/m² women; n (%)	90	30 (33.3)	76	13 (17.1)	0.0087		-16.2%	67	24 (35.8)	56	8 (14.2)	0.0034		-21.6%	23	6 (26.1)	20	5 (25.0)	0.467		-1.1%
Body cell mass, mean (SD), kg	78	28.2 (7.6)	63	34.4 (8.7)	<0.00001	5.2 (4.4)	21.1% (20.75)	57	30.0 (7.5)	51	36.5 (8.8)	<0.00001	5.8 (4.7)	22.3% (21.0)	21	23.3 (5.4)	16	27.7 (4.2)	<0.00001	3.6 (3.1)	17.3% (20.0)
SMMI, mean (SD), kg/m²	67	8.5 (2.4)	59	9.6 (3.8)	<0.00001	1.4 (3.5)	19.1% (48.5)	47	8.9 (2.0)	43	9.7 (2.3)	<0.00001	0.9 (1.1)	12.3% (12.5)	20	7.4 (2.9)	16	9.3 (6.4)	0.0001	2.5 (6.4)	36.5% (88.7)
Total body water, mean (SD), L	86	39.3 (8.4)	79	43.4 (9.0)	<0.00001	4.3 (4.4)	11.5% (11.0)	64	42.1 (7.5)	59	46.7 (7.7)	<0.00001	4.8 (4.8)	12.3% (11.8)	22	31.1 (4.6)	20	33.6 (3.8)	0.0001	2.6 (2.1)	9.1% (7.4)

ECW, mean (SD), L	88	18.5 (5.4)	81	18.9 (4.6)	0.0010	0.6 (2.0)	5.2% (10.9)	64	19.9 (5.3)	61	20.3 (4.3)	0.036	0.8 (2.2)	5.7% (11.5)	24	14.9 (3.8)	20	14.6 (2.5)	0.1055	-0.3 (1.3)	-3.5 (8.9)
ICW, mean (SD), L	82	20.6 (5.1)	69	24.5 (5.3)	<0.00001	3.6 (3.0)	19.1% (17.8)	60	22.1 (4.7)	53	26.0 (5.0)	<0.00001	4.0 (3.2)	20.0% (18.3)	22	16.5 (3.4)	16	19.6 (2.8)	0.0001	2.5 (2.0)	16.2% (16.3)
ASMM index, mean (SD), kg/m²	49	7.2 (2.1)	41	7.7 (1.8)	0.0002	0.6 (1.8)	11.4% (20.0)	36	7.3 (2.1)	32	7.9 (1.9)	0.0013	0.6 (1.9)	11.8% (21.6)	13	7.03 (2.1)	9	7.1 (1.5)	0.14	0.6 (1.0)	10.0% (13.8)
ASMM index < 7 men or < 5.7 Kg/m² women, n (%)	49	20 (40.8)	41	12 (29.2)	0.127		-11.6%	36	17 (47.2)	32	10 (31.2)	0.0895		-16.0%	13	3 (23.0)	9	2 (22.2)	0.684		-0.8%
PhA, mean (SD), degrees	87	4.5 (1.0)	81	5.4 (0.9)	<0.00001	0.9 (0.7)	24.3% (22.2)	65	4.5 (1.1)	61	5.5 (1.0)	<0.00001	1.0 (0.8)	26.5% (23.4)	22	4.3 (0.9)	20	5.1 (0.8)	0.0007	0.7 (0.6)	17.4% (16.5)
PhA <3.95º	87	26 (29.8)	81	3 (3.7)	<0.0001		-26.1%	65	20 (30.7)	61	2 (3.2)	<0.0001		-27.5%	22	6 (27.2)	20	1 (5.0)	0.061		- 22.2%
Standardized PhA, median (IQR)	31	-2.0 (-2.7 to -1.0)	28	-1.2 (-1.8 to -0.7)	0.0071	0.5 (-0.2 to 1.7)	34.8% (-11 to 66)	23	-2.1 (-2.7 to -1.4)	22	-1.2 (-2 to -0.8)	0.0257	0.5 (-0.2 to 1.7)	33.3% (-11 to 62)	8	-1.4 (-3.5 to -0.7)	6	-1.1 (-1.4 to -0.4)	0.312	0.6 (0.4 to 2.1)	60% (21 to 77)
RZ, mean (SD), ohmios	55	529.3 (108.0)	49	465.2 (90.3)	<0.00001	-74.3 (77.7)	-12.8% (13.7)	43	519.2 (99.7)	39	454.2 (86.7)	<0.00001	-75.7 (75.4)	-13.3% (14.1)	12	565.3 (132.3)	10	508.0 (96.0)	0.077	-68.0 (92.7)	- 10.6% (12.6)
XC, mean (SD), ohmios	55	43.1 (11.3)	49	44.6 (9.2)	0.267	1.3 (8.0)	5.5% (19.5)	43	42.5 (10.8)	39	44.3 (9.0)	0.304	1.4 (8.2)	6.1% (20.3)	12	45.2 (13.0)	10	45.7 (10.5)	0.705	1.0 (7.7)	4.5 % (16.1)

Abbreviations: ASMM, appendicular skeletal muscle mass; ECW, extracellular water; FFMI, fat-free mass index; ICW, intracellular water, IQR, Interquartile range, PhA, phase angle; RZ: raw resistance; SMMI, skeletal muscle mass index; XC: reactance.

Table S6. Correlations between the percentage change after the intervention of variables measured in BIA and ultrasound.

		**BIA measures**: Percentage change after 90 days of intervention										
		Weight, kg	Fat mass, kg	Fat-free mass, kg	FFMI, kg/m²	Body cell mass, kg	SMMI, kg/m²	ASMM index, kg/m²	PhA, degrees	Standardized PhA	RZ, ohmios	XC, ohmios
**US measures**: Percentage change after 90 days of intervention	**Rectus femoris**											
	RFCSA, cm²	n: 85 r: 0.169 p-value: 0.121	---	---	---	n: 63 r: 0.305 p-value: **0.015**	n: 53 r: 0.242 p-value: 0.080	n: 37 r: 0.403 p-value: **0.013**	---	n:26 r: -0.278 p-value: 0.169	---	n: 45 r: 0.251 p-value: 0.095
	Muscle circumference, cm	n: 85 r: 0.378 p-value: **0.0004**	n: 81 r: 0.250 p-value: **0.024**	---	---	---	---	---	---	---	---	---
	X-axis, cm	---	---	---	---	n: 63 r: 0.311 p-value: **0.012**	---	---	---	---	---	n: 45 r: 0.258 p-value: 0.086
	Y-axis, cm	n: 85 r: 0.273 p-value: **0.011**	n: 81 r: 0.222 p-value: **0.045**	---	---	---	n: 53 r: 0.254 p-value: 0.065	n: 37 r: 0.347 p-value: **0.034**	---	---	n: 45 r: 0.227 p-value: 0.132	n: 45 r: 0.338 p-value: **0.023**
	Subcutaneous adipose tissue, cm	---	---	---	---	---	---	---	---	---	---	---
	**Abdominal wall**											

	Total adipose tissue, cm	n: 84<br>r: 0.405<br>p-value: **<0.0001**	n: 80<br>r: 0.272<br>p-value: **0.014**	n: 71<br>r: 0.182<br>p-value: 0.128	n: 71<br>r: 0.171<br>p-value: 0.152	---	---	---	---	---	---	---
	Superficial adipose tissue, cm	n: 84<br>r: 0.167<br>p-value: 0.128	n: 80<br>r: 0.177<br>p-value: 0.115	---	---	---	---	---	n: 75<br>r: -0.187<br>p-value: 0.108	---	---	---
	Preperitoneal adipose tissue, cm	n: 84<br>r: 0.268<br>p-value: **0.0134**	n: 80<br>r: 0.296<br>p-value: **0.007**	---	---	---	---	---	---	---	---	n: 45<br>r: 0.261<br>p-value: 0.082

Abbreviations: ASMM, appendicular skeletal muscle mass; FFMI, fat-free mass index; PhA, phase angle; RFCSA, rectus femoris cross-sectional area, RZ: raw resistance; SMMI, skeletal muscle mass index; XC: reactance.

Only correlations with p <0.20 are shown.

Bold values denote statistical significance at the p < 0.05 level.

Table S7. Correlations between the percentage change after the intervention in BIA and ultrasound measurements and the percentage change in handgrip strength and TUG results.

		Percentage change after 90 days of intervention	
		**Handgrip strength, kg**	**TUG, seconds**
**BIA measures**: Percentage change after 90 days of intervention	Weight, kg	n: 79<br>r: 0.174<br>p-value: 0.124	n: 81<br>r: -0.170<br>p-value: 0.134
	Fat mass, kg	---	---
	Fat-free mass, kg	---	n: 70<br>r: -0.223<br>p-value: 0.062
	FFMI, kg/m^2	---	n: 70<br>r: -0.216<br>p-value: 0.071
	Body cell mass, kg	n: 62<br>r: 0.241<br>p-value: 0.058	n: 62<br>r: -0.190<br>p-value: 0.139
	SMMI, kg/m^2	---	n: 52<br>r: -0.306<br>**p-value: 0.026**
	ASMM index, kg/m^2	---	---
	PA, degrees	n: 74<br>r: 0.335<br>**p-value: 0.0034**	n: 74<br>r: -0.264<br>**p-value: 0.022**
	Standardized PhA	n: 25<br>r: -0.305<br>p-value: 0.137	---
	RZ, ohmios	---	n: 45<br>r: 0.311<br>**p-value: 0.042**
	XC, ohmios	n: 44<br>r: 0.363<br>**p-value: 0.0152**	n: 45<br>r: 0.205<br>p-value: 0.185
**US measurements**: Percentage change after 90 days of intervention	**Rectus femoris**		
	RFCSA, cm^2	n: 83<br>r: 0.302<br>**p-value: 0.005**	n: 83<br>r: -0.192<br>p-value: 0.081
	Muscle circumference, cm	n: 83<br>r: 0.196<br>p-value: 0.075	---
	X-axis, cm	n: 83<br>r: 0.279	---

		**p-value: 0.010**	
	Y-axis, cm	n: 83<br>r: 0.248<br>**p-value: 0.023**	n: 83<br>r: -0.225<br>**p-value: 0.040**
	Subcutaneous adipose tissue, cm	---	---
	**Abdominal wall**		
	Total adipose tissue, cm	---	---
	Superficial adipose tissue, cm	---	---
	Preperitoneal adipose tissue, cm	---	---

Abbreviations: ASMM, appendicular skeletal muscle mass; FFMI, fat-free mass index; PhA, phase angle; RFCSA, rectus femoris cross-sectional area; RZ: raw resistance; SMMI, skeletal muscle mass index; XC: reactance.

Only correlations with $p < 0.20$ are shown.

Bold values denote statistical significance at the $p < 0.05$ level.

Table S8. Tolerance after 45 and 90 days of the intervention.

	Day 45 (n = 90)											Day 90 (n = 85)										
	Yes						No					Yes					No					
Any discomfort/side effect, n (%)	26 (28.8)						64 (71.1)					18 (21.1)					67 (78.8)					
**(0: very bad - 10: very good)**	**0**	**1**	**2**	**3**	**4**	**5**	**6**	**7**	**8**	**9**	**10**	**0**	**1**	**2**	**3**	**4**	**5**	**6**	**7**	**8**	**9**	**10**
How have you been feeling during the last 7 days? n (%)	1 (1.1)	0	0	0	1 (1.1)	8 (8.8)	9 (10.0)	20 (22.2)	24 (26.6)	16 (17.7)	11 (12.2)	3 (3.5)	0	0	0	2 (2.3)	4 (4.7)	6 (7.0)	18 (21.1)	19 (22.3)	12 (14.1)	21 (24.7
**(0: not at all - 10: very frequently) ***	**0**	**1**	**2**	**3**	**4**	**5**	**6**	**7**	**8**	**9**	**10**	**0**	**1**	**2**	**3**	**4**	**5**	**6**	**7**	**8**	**9**	**10**
During the last 7 days you suffered from: n (%)																						
Nausea?	84 (94.3)	3 (3.3)	1 (1.1)	1 (1.1)	0	0	0	0	0	0	0	76 (89.4)	3 (3.5)	1 (1.1)	0	0	0	2 (2.35)	2 (2.35)	0	0	1 (1,1)
Vomiting?	87 (97.7)	2 (2.2)	0	0	0	0	0	0	0	0	0	83 (97.6)	0	0	0	0	0	0	1 (1.1)	0	0	1 (1.1)
Diarrhea?	83 (93.2)	1 (1.1)	1 (1.1)	0	1 (1.1)	0	0	2 (2.2)	1 (1.1)	0	0	75 (88.2)	2 (2.3)	2 (2.3)	1 (1.1)	0	1 (1.1)	1 (1.1)	1 (1.1)	1 (1.1)	0	1 (1.1)
Constipation?	67 (75.2)	3 (3.3)	3 (3.3)	3 (3.3)	2 (2.2)	2 (2.2)	2 (2.2)	3 (3.3)	1 (1.1)	2 (2.2)	1 (1.1)	64 (75.2)	2 (2.3)	2 (2.3)	3 (3.5)	1 (1.1)	1 (1.1)	2 (2.3)	3 (3.5)	4 (4.7)	2 (2.3)	1 (1.1)
Reflux or heartburn?	76 (85.3)	1 (1.1)	3 (3.3)	1 (1.1)	1 (1.1)	2 (2.2)	3 (3.3)	1 (1.1)	0	0	1 (1.1)	68 (80.0)	4 (4.7)	1 (1.1)	0	0	2 (2.3)	5 (5.8)	3 (3.5)	1 (1.1)	0	1 (1.1)
Abdominal pain?	76 (85.3)	4 (4.4)	4 (4.4)	0	1 (1.1)	1 (1.1)	0	2 (2.2)	1 (1.1)	0	0	74 (87.0)	2 (2.3)	3 (3.5)	0	0	1 (1.1)	2 (2.3)	2 (2.3)	0	0	1 (1.1)
Bloating?	63 (70.7)	1 (1.1)	4 (4.4)	1 (1.1)	3 (3.3)	4 (4.4)	3 (3.3)	5 (5.6)	2 (2.2)	3 (3.3)	0	60 (70.5)	2 (2.3)	4 (4.7)	1 (1.1)	7 (8.2)	1 (1.1)	1 (1.1)	6 (1.1)	1 (1.1)	0	2 (2.3)
Stomachache?	73 (82.0)	4 (4.4)	3 (3.3)	2 (2.2)	0	4 (4.4)	1 (1.1)	1 (1.1)	1 (1.1)	0	0	71 (83.5)	4 (4.7)	4 (4.7)	1 (1.1)	0	1 (1.1)	1 (1.1)	1 (1.1)	1 (1.1)	0	1 (1.1)
Flatulence?	56 (62.9)	4 (4.4)	5 (5.6)	7 (7.8)	2 (2.2)	2 (2.2)	5 (5.6)	3 (3.3)	2 (2.2)	2 (2.2)	1 (1.1)	55 (64.7)	1 (1.1)	3 (3.5)	3 (3.5)	3 (3.5)	3 (3.5)	3 (3.5)	3 (3.5)	5 (5.8)	3 (3.5)	3 (3.5)
During the last 7 days, have you felt full after taking the supplement?	22 (24.7)	1 (1.1)	5 (5,6)	5 (5,6)	4 (4.4)	10 (11.2)	4 (4.4)	10 (11.2)	12 (13.4)	5 (5,6)	11 (12.3)	21 (24.7)	2 (2.3)	5 (5.8)	0	4 (4.7)	13 (15.2)	8 (9.4)	7 (8.2)	11 (12.9)	2 (2.3)	12 (14.1)

*Data from 89 patients on day 45 and 85 on day 90.
